# Circulating NLRP3 Inflammasome Activation Is Associated With Sepsis‐Associated Acute Kidney Injury and Disease Severity: A Prospective Pilot Study

**DOI:** 10.1002/iid3.70559

**Published:** 2026-10-26

**Authors:** Xiaoqian Zhang, Wang Zhang, Ju Guo, Mikeriayi Aihemaiti, Yunlin Song

**Affiliations:** ^1^ Department of Critical Care Medicine The First Affiliated Hospital of Xinjiang Medical University Urumqi China; ^2^ Gastrointestinal Surgery Department People's Hospital of Xinjiang Uygur Autonomous Region Urumqi China; ^3^ Xinjiang International Medical Center (Xinjiang International Hospital Xinjiang Academy of Medical Sciences) Urumqi China

**Keywords:** biomarker, disease severity, NLRP3 inflammasome, sepsis‐associated acute kidney injury, systemic inflammation

## Abstract

**Background:**

Sepsis‐associated acute kidney injury (SA‐AKI) is a severe and prevalent complication in critically ill patients, characterized by high mortality and a lack of reliable biomarkers that reflect inflammatory pathway activation and disease severity. Although the NLRP3 inflammasome is known to mediate inflammatory responses, pyroptosis, and renal injury, its clinical relevance in human SA‐AKI remains poorly defined. This study aimed to characterize the expression profiles, dynamic trajectories, and clinical associations of circulating NLRP3 inflammasome components in patients with SA‐AKI.

**Materials and Methods:**

Thirty adult patients with sepsis (13 with SA‐AKI and 17 without AKI) were prospectively and consecutively enrolled. Sepsis was defined according to the Sepsis‐3 consensus criteria, whereas AKI was diagnosed and staged exclusively using the Kidney Disease: Improving Global Outcomes (KDIGO) criteria. Serum protein levels of NLRP3, ASC, caspase‐1, cleaved caspase‐1, IL‐1β, and IL‐18, and transcript levels of NLRP3, ASC, and caspase‐1, were determined on days 1, 3, 5, and 7 after enrollment using ELISA and quantitative real‐time PCR. Statistical analyses included group comparisons, Spearman rank correlations, linear mixed‐effects models, and exploratory receiver operating characteristic (ROC) analyses to assess concurrent group discrimination. Because samples were collected post‐classification, these analyses describe post‐diagnostic expression kinetics rather than prediction of future AKI onset.

**Results:**

The expression of NLRP3 inflammasome‐related molecules was significantly upregulated in the SA‐AKI group and correlated with renal dysfunction and elevated illness‐severity scores (SOFA and APACHE II). Exploratory ROC analyses suggested strong discrimination of concurrent AKI status for ASC, caspase‐1, and NLRP3 transcript levels, with the strongest overall performance observed on day 3. These estimates are hypothesis‐generating because of the small sample size and post‐classification sampling.

**Discussion:**

The NLRP3 inflammasome pathway is prominently activated in patients with SA‐AKI and is closely associated with renal dysfunction and systemic illness severity. Circulating components of this pathway are candidate biomarkers of concurrent SA‐AKI, but their ability to predict AKI onset or prognosis requires validation in larger prospective cohorts sampled before AKI develops.

## 1 Introduction

Sepsis‐associated acute kidney injury (SA‐AKI) refers to AKI occurring in the context of sepsis. Sepsis is defined according to the Third International Consensus Definitions for Sepsis and Septic Shock (Sepsis‐3) [1], and AKI is diagnosed and staged according to the Kidney Disease: Improving Global Outcomes (KDIGO) criteria [2]. SA‐AKI is the most common cause of AKI in critically ill patients: approximately 30%–70% of septic patients develop AKI, contributing to an estimated 6–11 million new SA‐AKI cases globally each year [3, 4, 5]. Within the intensive care unit (ICU) setting, the incidence of SA‐AKI ranges from 47.1% to 54%, accounting for nearly half of all AKI episodes [6, 7]. In China, a multicenter retrospective study of hospitalized adults placed the incidence of AKI among septic patients at 47.1% [7]. SA‐AKI severely worsens patient prognoses, prolongs hospital stays, and increases healthcare costs [3, 4]. Consequently, biomarkers that accurately reflect renal injury and systemic inflammatory burden may improve the clinical assessment of these critically ill patients; however, established biomarkers remain insufficiently sensitive and specific for this purpose [8].

Accumulating evidence indicates that hyperinflammatory responses, cellular apoptosis, and microvascular dysfunction drive the pathogenesis of AKI, with inflammasome activation serving as a crucial upstream mediator [5, 9]. Among these complexes, the NLRP3 inflammasome—named for its sensor component NLRP3 (NOD‐, LRR‐ and pyrin domain‐containing protein 3)—is the most extensively studied. It comprises the intracellular sensor NLRP3, the adaptor protein ASC (apoptosis‐associated speck‐like protein containing a caspase recruitment domain [CARD]), and the effector protease caspase‐1 [9]. Upon detecting pathogen‐associated or damage‐associated molecular patterns (PAMPs/DAMPs), NLRP3 promotes complex assembly and autoproteolytic activation of caspase‐1, which in turn drives the maturation and secretion of the pro‐inflammatory cytokines interleukin (IL)−1β and IL‐18 and concurrently executes pyroptotic cell death [10, 11]. Although local tissue activation of the NLRP3 axis has been documented to aggravate intrarenal inflammation in experimental kidney injury [12, 13], its systemic expression kinetics and clinical associations in human SA‐AKI remain insufficiently defined [14]. We therefore hypothesized that circulating components of the NLRP3 inflammasome would be associated with the presence and severity of SA‐AKI.

To test this hypothesis, we conducted a prospective observational clinical study. We quantified dynamic serum protein and transcript levels of NLRP3, ASC, caspase‐1, cleaved caspase‐1, IL‐1β, and IL‐18 on days 1, 3, 5, and 7 after enrollment in patients with sepsis. By comparing these trajectories between patients with SA‐AKI and non‐AKI septic controls, we aimed to characterize the longitudinal expression of this inflammasome axis and its associations with renal dysfunction and illness severity. Because biomarker sampling began after clinical classification, the present study does not evaluate prediction of future AKI onset.

## 2 Materials and Methods

### 2.1 Study Population and Diagnostic Criteria

We prospectively enrolled 30 adult patients diagnosed with sepsis in the intensive care unit. Sepsis was defined according to the Sepsis‐3 consensus criteria [1]. AKI was diagnosed and staged exclusively using the KDIGO criteria [2], based on changes in serum creatinine and/or urine output. Patients who met both the Sepsis‐3 definition of sepsis and the KDIGO definition of AKI were classified into the SA‐AKI group (*n* = 13). Patients who met the Sepsis‐3 definition but did not meet KDIGO AKI criteria during the observation period were classified as non‐AKI septic controls (*n* = 17). Sepsis‐3 criteria were not used to define AKI. Detailed demographic and clinical data were compiled at baseline, including age, sex, body mass index (BMI), pre‐existing comorbidities, APACHE II score, SOFA score, baseline and peak serum creatinine, baseline and peak blood urea nitrogen (BUN), and estimated glomerular filtration rate (eGFR). Baseline serum creatinine (SCr) was defined as the value measured at enrollment (day 1); peak SCr was defined as the highest value recorded during the 7‐day observation period, which occurred at a variable day across participants. BUN was recorded as a standard component of the routine renal panel. The study is reported in accordance with the Strengthening the Reporting of Observational Studies in Epidemiology (STROBE) guidelines [15] (S1 Checklist).

The study was reviewed by the Ethics Committee of the First Affiliated Hospital of Xinjiang Medical University, which granted an exemption from the requirement for full ethical review and, in line with this exemption, assigned no separate approval or reference number. The exemption was based on the study being a minimal‐risk observational investigation using routinely collected clinical data and residual diagnostic blood samples. The study was conducted in accordance with the principles of the Declaration of Helsinki. Because of this exemption, the Committee also waived the requirement for written informed consent; verbal informed consent was obtained from each patient or the patient's legally authorized surrogate at the time of enrollment.

### 2.2 Clinical Sample Collection and Biomarker Detection

Venous blood samples were collected on days 1, 3, 5, and 7 after enrollment in all participants according to an identical pre‐specified schedule; the interval between sepsis onset and enrollment was not standardized and may have differed among patients. For patients in the SA‐AKI group, AKI had already been identified by the KDIGO criteria before day‐1 sampling; therefore, all biomarker analyses describe post‐classification expression and concurrent group discrimination rather than prediction of incident AKI. The “peak” time point used in the analyses denotes the maximum value recorded during the observation period, which occurred at a variable day across participants. Serum was isolated via centrifugation at 3,000 × g for 15 min at 4°C and stored at −80°C. Total protein concentrations of NLRP3, ASC, caspase‐1, cleaved caspase‐1, IL‐1β, and IL‐18 were quantified using commercially available enzyme‐linked immunosorbent assay (ELISA) kits according to the manufacturers' instructions. Simultaneously, total RNA was extracted from peripheral blood mononuclear cells, and transcript levels of NLRP3, ASC, and caspase‐1 were measured using quantitative real‐time polymerase chain reaction (qRT‐PCR) with SYBR Green chemistry, with GAPDH as the internal reference control. Relative gene expression was calculated using the 2^(‐ΔΔCt)^ method. Primer sequences are available from the corresponding author upon request.

### 2.3 Statistical Analysis

Statistical evaluation was conducted using R software (version 4.4.1). Distribution normality was verified using the Shapiro–Wilk test. Normally distributed continuous parameters are reported as the mean ± standard deviation (mean ± SD) and were compared using the independent‐samples t‐test. Non‐normally distributed data are expressed as the median with interquartile range (median [IQR]) and were analyzed using the Wilcoxon rank‐sum test. Categorical variables are reported as frequencies with percentages (%) and were evaluated using the Chi‐square test or Fisher's exact test where appropriate.

Associations between biomarker expression and traditional indicators of renal injury were evaluated using Spearman's rank correlation coefficients, and the corresponding two‐tailed *p* values were computed for each comparison. Longitudinal variations in biomarker levels across the four predefined time points were analyzed using linear mixed‐effects models (LMM) to account for within‐subject correlation over time. Receiver operating characteristic (ROC) curves were constructed only as exploratory measures of concurrent group discrimination, with the area under the curve (AUC), sensitivity, and specificity calculated at optimal cutoff values determined by maximizing the Youden index. Given the small number of AKI events (*n* = 13) and the post‐classification sampling design, statistical modeling was strictly focused on exploratory longitudinal trends and bivariate associations. No multivariable prediction models, nomograms, or decision curves were constructed to avoid model overfitting. Two‐sided *p* values < 0.05 were considered statistically significant. Significance levels in tables are indicated as *p < 0.05, **p < 0.01, and ***p < 0.001.

## 3 Results

### 3.1 Baseline Characteristics

The clinical cohort comprised 30 patients with sepsis, categorized into an SA‐AKI group (*n* = 13) and a non‐AKI septic control group (*n* = 17) according to the KDIGO criteria. As detailed in Table 1, no significant differences were observed between the two groups regarding age, sex, BMI, or baseline chronic comorbidities (*p* > 0.05). Conversely, the SA‐AKI cohort demonstrated significantly elevated baseline and peak serum creatinine, baseline and peak BUN, higher APACHE II scores, prolonged ICU length of stay, and lower baseline eGFR compared with non‐AKI controls (*p* < 0.05). Baseline SOFA scores were comparable on day 1 (*p* > 0.05) but were significantly higher in the SA‐AKI group on days 3, 5, and 7 (*p* < 0.01).

**Table 1:** Baseline demographic and clinical characteristics of the study cohort, stratified by the presence or absence of sepsis‐associated acute kidney injury (SA‐AKI).

Characteristic	Non‐AKI sepsis (*n* = 17)	SA‐AKI (*n* = 13)	*p* value
Sex, *n* (%) Female	7 (41.2)	5 (38.5)	1.000
Male	10 (58.8)	8 (61.5)	
Age, years (mean ± SD)	57.1 ± 4.9	58.4 ± 13.3	0.719
BMI, kg/m^2^ (mean ± SD)	20.1 ± 1.2	20.6 ± 1.6	0.342
Comorbidity, *n* (%) No	9 (52.9)	5 (38.5)	0.676
Yes	8 (47.1)	8 (61.5)	
Baseline SCr, μmol/L, median [IQR]	80.0 [74.0, 86.0]	157.0 [136.0, 189.0]	< 0.001
Peak SCr, μmol/L, median [IQR]	94.0 [89.0, 102.0]	370.0 [328.0, 405.0]	< 0.001
Baseline BUN, mmol/L (mean ± SD)	6.1 ± 2.5	8.4 ± 1.9	0.011
Peak BUN, mmol/L, median [IQR]	7.3 [5.7, 10.8]	12.5 [10.6, 13.6]	0.004
eGFR, mL/min/1.73 m^2^, median [IQR]	73.4 [65.8, 74.9]	13.6 [12.4, 16.4]	< 0.001
APACHE II, median [IQR]	15.0 [13.0, 16.0]	24.0 [19.0, 28.0]	< 0.001
AKI duration, days, median [IQR]	0.0 [0.0, 0.0]	2.0 [1.0, 3.0]	< 0.001
Total ICU LOS, days (mean ± SD)	9.0 ± 3.7	12.5 ± 2.6	0.008
SOFA day 1 (mean ± SD)	9.9 ± 2.8	11.9 ± 3.4	0.087
SOFA day 3 (mean ± SD)	7.4 ± 3.4	14.2 ± 3.2	< 0.001
SOFA day 5 (mean ± SD)	6.2 ± 2.6	11.8 ± 4.0	< 0.001
SOFA day 7 (mean ± SD)	5.1 ± 1.6	8.9 ± 3.7	0.001

*Note:* Data are presented as *n* (%), mean ± SD, or median [IQR]. *p* values were calculated using the Chi‐square test or Fisher's exact test (categorical variables), the independent‐samples t‐test (normally distributed continuous variables), or the Wilcoxon rank‐sum test (non‐normally distributed continuous variables).

Abbreviations: AKI, acute kidney injury; APACHE II, Acute Physiology and Chronic Health Evaluation II; BMI, body mass index; BUN, blood urea nitrogen; eGFR, estimated glomerular filtration rate; ICU, intensive care unit; IQR, interquartile range; LOS, length of stay; SCr, serum creatinine; SD, standard deviation; SOFA, Sequential Organ Failure Assessment.

### 3.2 Expression of Inflammasome‐Related Molecules

The expression levels of the six evaluated markers—NLRP3, ASC, caspase‐1, cleaved caspase‐1, IL‐1β, and IL‐18—measured by ELISA (protein) and, for NLRP3, ASC, and caspase‐1, by qRT‐PCR (transcript), are presented in Figures 1 and 2. Protein and transcript levels of all measured markers were consistently higher in the SA‐AKI cohort than in the non‐AKI cohort across the tracked time points; group differences at each time point were evaluated using the Wilcoxon rank‐sum test (Section 3.6; Table 8), and longitudinal trends were formally analyzed using linear mixed‐effects models (Section 3.4; Tables 4 and 5).

**Figure 1:**
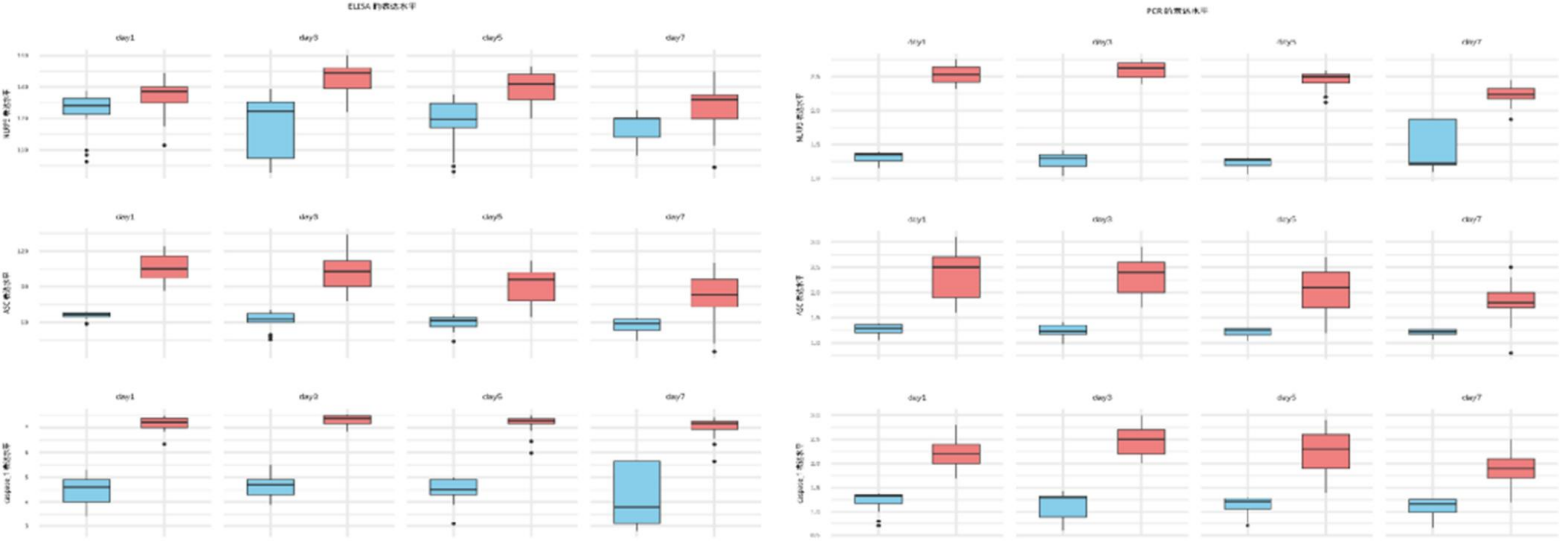
Box plots of serum protein (ELISA) and transcript (qRT‐PCR) levels of NLRP3, ASC, caspase‐1, cleaved caspase‐1, IL‐1β, and IL‐18 at days 1, 3, 5, and 7 and at the peak value in patients with SA‐AKI (*n* = 13) and non‐AKI septic controls (*n* = 17). Boxes show the median and interquartile range; whiskers show the range; outliers are shown as individual points. Group labels and significance markers are indicated within each panel. *p < 0.05, **p < 0.01, ***p < 0.001 (Wilcoxon rank‐sum test).

**Figure 2:**
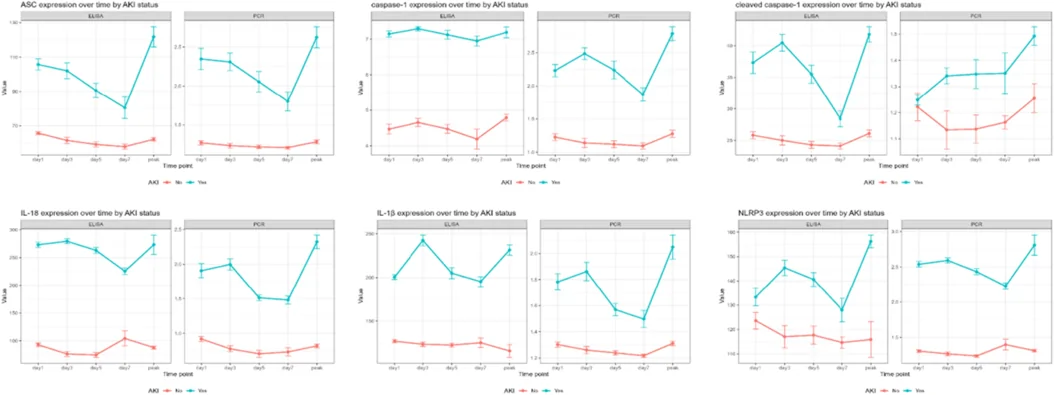
Longitudinal trends in serum protein (ELISA) and transcript (qRT‐PCR) levels of NLRP3, ASC, caspase‐1, cleaved caspase‐1, IL‐1β, and IL‐18 at days 1, 3, 5, and 7 and at the peak value in patients with SA‐AKI and non‐AKI septic controls. Data are shown as the mean ± SD. The “peak” point denotes the maximum value recorded during the 7‐day observation period, which occurred at a variable day across participants; it is plotted separately and should not be interpreted as a fixed time point. Group differences at each time point were tested using the Wilcoxon rank‐sum test. *p < 0.05, **p < 0.01, ***p < 0.001.

### 3.3 Correlations of Inflammasome Components With Renal and Clinical Indicators

As detailed in Tables 2 and 3, serum and transcript levels of NLRP3 correlated significantly with baseline creatinine (rho = 0.4–0.7) and inversely with eGFR across multiple time points. Transcript measurements obtained via qRT‐PCR exhibited higher stability and lower variance than ELISA protein profiles, particularly on days 1 and 7. Furthermore, NLRP3 expression displayed a strong positive correlation with concurrent SOFA scores, reaching a peak Spearman's rho of 0.977 on day 3 (Tables 2 and 3), highlighting its alignment with overall illness severity. BUN was included because it is a standard component of the renal panel recorded in routine ICU care; its correlations with the inflammasome markers were weaker than those of SCr and eGFR (Tables 2 and 3), suggesting that the associations are strongest with direct markers of glomerular filtration.

**Table 2:** Correlations of ELISA measurements with clinical indicators on days 1, 3, 5, and 7 and at peak creatinine.

	NLRP3	ASC	IL‐18	IL‐1β	Cleaved caspase‐1	Caspase‐1
Baseline creatinine	0.60–0.76	0.56–0.80	0.74–0.79	0.75–0.78	0.68–0.76	0.71–0.76
Peak creatinine	0.50–0.79	0.62–0.81	0.76–0.83	0.77–0.81	0.63–0.79	0.75–0.81
eGFR	−0.4 to −0.60	−0.58 to −0.70	−0.60 to −0.80	−0.60 to −0.79	−0.54 to −0.64	−0.62 to −0.81
Baseline BUN	0.38–0.47	0.40–0.47	0.42–0.57	0.42–0.55	0.38–0.51	0.36–0.49
Peak BUN	0.40–0.60	0.37–0.53	0.48–0.58	0.49–0.56	0.45–0.52	0.42–0.54
APACHE II	0.58–0.82	0.61–0.82	0.65–0.82	0.53–0.83	0.56–0.83	0.61–0.90
SOFA score on day 1	0.57–0.97	0.50–0.69	0.43–0.73	0.42–0.73	0.53–0.71	0.41–0.65
SOFA score on day 3	0.68–0.98	0.66–0.95	0.66–0.97	0.66–0.96	0.67–0.96	0.56–0.91
SOFA score on day 5	0.74–0.95	0.75–0.94	0.67–0.92	0.72–0.89	0.77–0.97	0.57–0.77
SOFA score on day 7	0.61–0.90	0.61–0.97	0.55–0.86	0.61–0.84	0.62–0.98	0.44–0.71

*Note:* Values are Spearman's rank correlation coefficients (rho), shown as ranges across the four time points and the peak value. *p< 0.05; **p< 0.01; ***p< 0.001 (two‐tailed). The complete per‐comparison correlation matrix with P values is provided in Supplementary Table S3. Abbreviations: BUN, blood urea nitrogen; eGFR, estimated glomerular filtration rate; APACHE II, Acute Physiology and Chronic Health Evaluation II; SOFA, Sequential Organ Failure Assessment.

**Table 3:** Correlations of PCR measurements with clinical indicators on days 1, 3, 5, and 7 and at peak creatinine.

	NLRP3	ASC	IL‐18	IL‐1β	Cleaved caspase‐1	Caspase‐1
Baseline creatinine	0.66–0.72	0.61–0.78	0.75–0.85	0.51–0.78	0.33–0.51	0.70–0.82
Peak creatinine	0.68–0.74	0.70–0.80	0.76–0.90	0.60–0.80	0.36–0.59	0.74–0.83
eGFR	−0.69 to −0.84	−0.61 to −0.70	−0.60 to −0.79	−0.61 to −0.70	−0.42 to −0.59	−0.63 to −0.73
Baseline BUN	0.34–0.44	0.36–0.43	0.42–0.58	0.31–0.42	−0.25 to 0.38	0.37–0.49
Peak BUN	0.68–0.74	0.70–0.80	0.77–0.90	0.60–0.80	0.38–0.59	0.74–0.83
APACHE II	0.52–0.88	0.49–0.84	0.73–0.83	0.36–0.83	0.48–0.59	0.54–0.85
SOFA score on day 1	0.43–0.62	0.57–0.71	0.42–0.74	0.39–0.70	0.38–0.94	0.41–0.67
SOFA score on day 3	0.52–0.85	0.62–0.96	0.66–0.96	0.60–0.96	0.36–0.86	0.55–0.92
SOFA score on day 5	0.56–0.81	0.72–0.93	0.69–0.92	0.72–0.91	0.57–0.91	0.59–0.79
SOFA score on day 7	0.54–0.65	0.61–0.88	0.59–0.85	0.61–0.89	0.51–0.78	0.52–0.72

*Note:* Values are Spearman's rank correlation coefficients (rho), shown as ranges across the four time points and the peak value. *p< 0.05; **p< 0.01; ***p< 0.001 (two‐tailed). The complete per‐comparison correlation matrix with P values is provided in Supplementary Table S3. Abbreviations: BUN, blood urea nitrogen; eGFR, estimated glomerular filtration rate; APACHE II, Acute Physiology and Chronic Health Evaluation II; SOFA, Sequential Organ Failure Assessment.

Extended analysis (Tables 2 and 3) confirmed that all core inflammasome elements (NLRP3, ASC, caspase‐1, and cleaved caspase‐1) maintained sustained correlations (rho > 0.3) with standard renal markers and severity indices; the corresponding two‐tailed *p* values for each comparison are provided in the complete correlation matrices (Supplementary Table S3). The correlations between ASC, NLRP3, and caspase‐1 levels and sequential SOFA scores are visually aggregated in Figure 3A–F.

**Figure 3:**
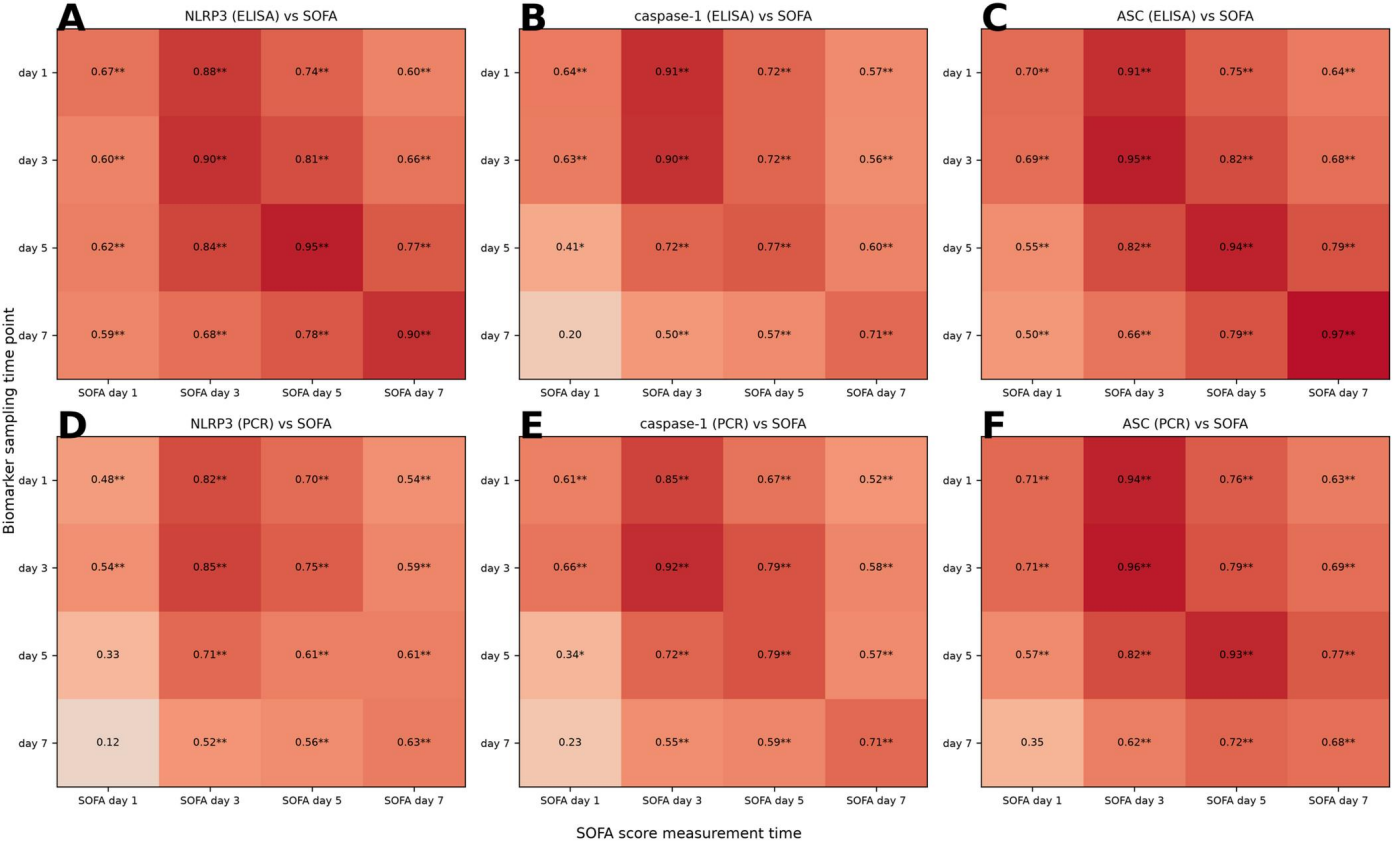
Heatmaps of Spearman's rank correlation coefficients between NLRP3, ASC, and caspase‐1 expression (protein, ELISA; transcript, qRT‐PCR) at each sampling time point and SOFA scores measured on days 1, 3, 5, and 7. (A–C) ELISA measurements of NLRP3, caspase‐1, and ASC, respectively. (D–F) qRT‐PCR measurements of NLRP3, caspase‐1, and ASC, respectively. The color scale indicates the strength of the correlation (rho). *p < 0.05, **p < 0.01 (two‐tailed).

### 3.4 Linear Mixed‐Effects Model for NLRP3

For NLRP3 protein levels measured by ELISA, the linear mixed model identified a significant fixed effect for time (β = 2.00, *p*< 0.001), indicating a progressive systemic rise across the cohort. AKI status and the time‐by‐AKI interaction did not reach independent significance; the concurrent SOFA score emerged as a strong positive covariate (β = 4.19, *p*< 0.001; Table 4). The absence of an independent effect of AKI status in this model is likely explained by the strong collinearity between AKI status and SOFA score in this cohort, rather than by a lack of discrimination: univariate group comparisons (Table 8) and exploratory ROC analyses (Tables 6 and 7) demonstrated clear group differences.

**Table 4:** Linear mixed‐effects model analysis of NLRP3 protein levels measured by ELISA.

Effect	Estimate	Standard error	t value	*p* value	Sig.
Intercept	80.25	2.80	28.66	< 0.001	***
Time	2.00	0.37	5.35	< 0.001	***
AKI status	2.12	2.67	0.79	0.430	ns
SOFA score	4.19	0.22	19.27	< 0.001	***
Time × AKI status	−0.67	0.51	−1.31	0.193	ns

*Note:* Sig., significance: *p < 0.05; **p < 0.01; ***p < 0.001; ns, not significant. The outcome variable is the serum NLRP3 protein level (ELISA).

For NLRP3 transcript levels measured by PCR, time maintained a significant positive association (β = 0.042, *p*< 0.001; Table 5), and AKI status showed a strong independent baseline effect (β = 1.275, *p* < 0.001). The time‐by‐AKI interaction term was significantly negative (β = −0.075, *p* < 0.001). This interaction reflects a divergence in expression over time: transcript levels in the SA‐AKI group gradually stabilized or decreased slightly after an early peak, whereas those in the non‐AKI group showed a modest late increase. The inclusion of the SOFA score remained highly significant in the RNA model (β = 0.038, *p* < 0.001).

**Table 5:** Linear mixed‐effects model analysis of NLRP3 mRNA levels measured by PCR.

Effect	Estimate	Standard error	t value	*p* value	Sig.
Intercept	0.864	0.070	12.34	< 0.001	***
Time	0.042	0.008	5.15	< 0.001	***
AKI status	1.275	0.067	18.98	< 0.001	***
SOFA score	0.038	0.005	6.94	< 0.001	***
Time × AKI status	−0.075	0.011	−7.07	< 0.001	***

*Note:* Sig., significance: *p < 0.05; **p < 0.01; ***p < 0.001; ns, not significant. The outcome variable is the NLRP3 transcript level (qRT‐PCR).

### 3.5 Exploratory ROC Analysis of ASC, NLRP3, and Caspase‐1

Exploratory ROC analyses were used to assess discrimination of concurrent SA‐AKI status (Tables 6 and 7; Figure 4A–F). Both serum and transcript levels of ASC and caspase‐1 showed complete group separation (AUC = 1.000) on days 1 and 3. For NLRP3, qRT‐PCR tracking maintained complete separation (AUC = 1.000) from days 1 through 5. In contrast, NLRP3 ELISA protein assessment showed lower discrimination on day 1 (AUC = 0.742) but higher discrimination on days 3 and 5 (AUC > 0.90). The computed optimal cutoffs exhibited a gradual downward trend over the 7‐day observation period. Because all measurements were obtained after clinical classification, these AUC values do not represent prediction of future AKI onset and should be interpreted cautiously given the small sample size and complete separation.

**Table 6:** Receiver operating characteristic (ROC) analysis of protein expression levels of the three molecules for discrimination of concurrent AKI.

Molecule	Time point	AUC	95% CI	Optimal cutoff	Sensitivity	Specificity
ASC	Day 1	1.000	1.000–1.000	77.800	1.000	1.000
	Day 3	1.000	1.000–1.000	74.200	1.000	1.000
	Day 5	0.982	0.943–1.000	67.600	0.923	1.000
	Day 7	0.846	0.642–1.000	63.900	0.846	1.000
NLRP3	Day 1	0.742	0.547–0.937	136.015	0.538	0.941
	Day 3	0.919	0.813–1.000	138.825	0.769	1.000
	Day 5	0.914	0.814–1.000	136.160	0.692	1.000
	Day 7	0.769	0.561–0.977	127.655	0.692	1.000
caspase‐1	Day 1	1.000	1.000–1.000	5.820	1.000	1.000
	Day 3	1.000	1.000–1.000	6.175	1.000	1.000
	Day 5	1.000	1.000–1.000	5.490	1.000	1.000
	Day 7	0.989	0.965–1.000	5.985	0.923	1.000

*Note:* AUC, area under the curve; CI, confidence interval. AUC values reflect discrimination of AKI status that was already present at the time of sampling (concurrent SA‐AKI), not prediction of future AKI onset.

**Table 7:** Receiver operating characteristic (ROC) analysis of transcript expression levels of the three molecules for discrimination of concurrent AKI.

Molecule	Time point	AUC	95% CI	Optimal cutoff	Sensitivity	Specificity
ASC	Day 1	1.000	1.000–1.000	1.495	1.000	1.000
	Day 3	1.000	1.000–1.000	1.555	1.000	1.000
	Day 5	0.957	0.871–1.000	1.350	0.923	1.000
	Day 7	0.923	0.772–1.000	1.285	0.923	1.000
NLRP3	Day 1	1.000	1.000–1.000	1.855	1.000	1.000
	Day 3	1.000	1.000–1.000	1.900	1.000	1.000
	Day 5	1.000	1.000–1.000	1.710	1.000	1.000
	Day 7	0.989	0.965–1.000	1.945	0.923	1.000
Caspase‐1	Day 1	1.000	1.000–1.000	1.540	1.000	1.000
	Day 3	1.000	1.000–1.000	1.715	1.000	1.000
	Day 5	1.000	1.000–1.000	1.350	1.000	1.000
	Day 7	0.966	0.897–1.000	1.330	0.923	1.000

*Note:* AUC, area under the curve; CI, confidence interval. AUC values reflect discrimination of AKI status that was already present at the time of sampling (concurrent SA‐AKI), not prediction of future AKI onset.

**Figure 4:**
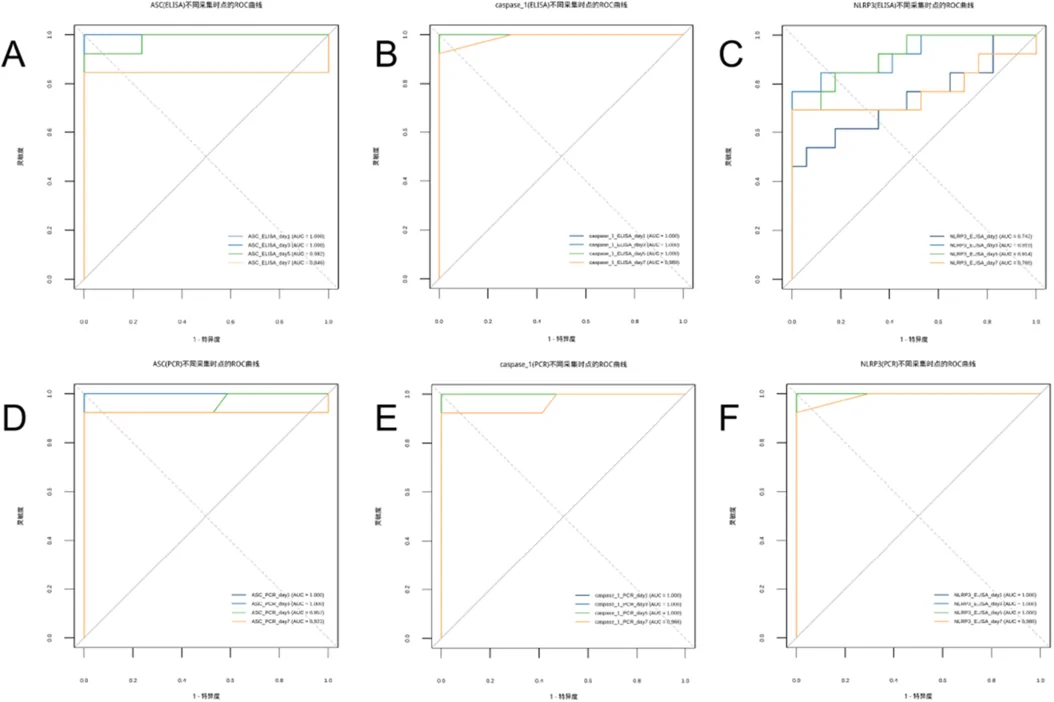
Receiver operating characteristic (ROC) curves for serum protein (ELISA) and transcript (qRT‐PCR) levels of ASC, caspase‐1, and NLRP3 for discrimination of concurrent SA‐AKI at days 1, 3, 5, and 7. (A–C) ELISA measurements of ASC, caspase‐1, and NLRP3, respectively. (D–F) qRT‐PCR measurements of ASC, caspase‐1, and NLRP3, respectively. AUC, area under the curve.

### 3.6 Group Comparisons Using the Wilcoxon Rank‐Sum Test

The Wilcoxon rank‐sum test confirmed that the expression levels of ASC, NLRP3, and caspase‐1 differed significantly between the two groups at every time point (adjusted *p*< 0.05; Table 8). IL‐18 and IL‐1β protein levels were consistently and significantly higher in the SA‐AKI group across all time points (Figures 1 and 2); IL‐18 and IL‐1β protein levels were consistently and significantly higher in the SA‐AKI group at all time points (all adjusted *p* < 0.001; Figures 1 and 2; Table 8). Cleaved caspase‐1 protein levels were consistently elevated across all time points, whereas its transcript differential expression reached statistical significance from day 5 onward, consistent with the group‐separation trends observed in the exploratory ROC analysis. Taken together, these univariate and group comparison analyses confirm a robust, highly synchronized upregulation of circulating NLRP3 inflammasome components in patients with SA‐AKI, which strongly tracks with disease severity scores and concurrent renal dysfunction.

**Table 8:** Wilcoxon rank‐sum test results for the expression levels of each molecule at different time points.

Molecule	Time point	ELISA			PCR		
		Raw *p*	Adj. *p* value	Sig.	Raw *p*	Adj. *p* value	Sig.
ASC	Day 1	< 0.001	< 0.001	***	< 0.001	< 0.001	***
	Day 3	< 0.001	< 0.001	***	< 0.001	< 0.001	***
	Day 5	< 0.001	< 0.001	***	< 0.001	< 0.001	***
	Day 7	0.001	0.002	**	< 0.001	< 0.001	***
	Peak	< 0.001	< 0.001	***	< 0.001	< 0.001	***
NLRP3	Day 1	0.027	0.027	*	< 0.001	< 0.001	***
	Day 3	< 0.001	< 0.001	***	< 0.001	< 0.001	***
	Day 5	< 0.001	< 0.001	***	< 0.001	< 0.001	***
	Day 7	0.013	0.014	*	< 0.001	< 0.001	***
	Peak	< 0.001	< 0.001	***	< 0.001	< 0.001	***
Caspase‐1	Day 1	< 0.001	< 0.001	***	< 0.001	< 0.001	***
	Day 3	< 0.001	< 0.001	***	< 0.001	< 0.001	***
	Day 5	< 0.001	< 0.001	***	< 0.001	< 0.001	***
	Day 7	< 0.001	< 0.001	***	< 0.001	< 0.001	***
	Peak	< 0.001	< 0.001	***	< 0.001	< 0.001	***
Cleaved caspase‐1	Day 1	< 0.001	< 0.001	***	0.240	0.240	ns
	Day 3	< 0.001	< 0.001	***	0.090	0.091	Marginal
	Day 5	< 0.001	< 0.001	***	0.013	0.014	*
	Day 7	0.002	0.002	**	0.010	0.011	*
	Peak	< 0.001	< 0.001	***	0.005	0.006	**
IL‐18	Day 1	<0.001	< 0.001	***	< 0.001	< 0.001	***
	Day 3	< 0.001	< 0.001	***	<0.001	< 0.001	***
	Day 5	< 0.001	< 0.001	***	< 0.001	< 0.001	***
	Day 7	< 0.001	< 0.001	***	< 0.001	< 0.001	***
	Peak	< 0.001	< 0.001	***	<0.001	< 0.001	***
IL‐1β	Day 1	< 0.001	< 0.001	***	< 0.001	< 0.001	***
	Day 3	< 0.001	< 0.001	***	< 0.001	< 0.001	***
	Day 5	< 0.001	< 0.001	***	<0.001	< 0.001	***
	Day 7	< 0.001	< 0.001	***	0.001	0.001	**
	Peak	< 0.001	< 0.001	***	< 0.001	< 0.001	***

*Note:* Raw *p* values were adjusted for multiple testing using the Benjamini–Hochberg method. Sig., significance: *p < 0.05; **p < 0.01; ***p < 0.001; ns, not significant; Marginal, 0.05 ≤ *p* < 0.10. Horizontal lines separate the individual analytes.

## 4 Discussion

In this prospective clinical cohort, circulating protein and transcript levels of core NLRP3 inflammasome components were significantly elevated in patients with SA‐AKI and correlated with renal dysfunction and overall illness severity. Exploratory ROC analyses showed strong separation between the SA‐AKI and non‐AKI groups for ASC, caspase‐1, and NLRP3 transcript levels, with the strongest overall associations observed on day 3. These findings characterize biomarker expression after clinical classification and should not be interpreted as evidence that the markers predict incident AKI.

The pathophysiological cascade of SA‐AKI is highly complex, involving microcirculatory dysfunction, metabolic reprogramming, oxidative stress, and hyperinflammation [16]. The NLRP3 inflammasome is an essential component of the innate immune response, aggregating in response to circulating PAMPs and DAMPs to catalyze caspase‐1 activation, which drives pyroptotic cell death and the maturation of IL‐1β and IL‐18 [9, 10, 11]. Our clinical data show a pronounced and sustained upregulation of these components in patients with SA‐AKI, consistent with previous preclinical findings that NLRP3 inflammasome activation contributes to experimental septic and other forms of AKI [12, 13, 17] and with a recent clinical study reporting elevated NLRP3 and IL‐18 in peripheral blood mononuclear cells of patients with SA‐AKI [14]. Cleaved caspase‐1, the direct enzymatic effector of this cascade, exhibited moderate negative correlations with eGFR (rho = −0.42 to −0.64 across time points; Tables 2 and 3), which—while not demonstrating causality—is consistent with its role as a marker of systemic inflammasome activation in the setting of reduced renal function.

A notable strength of this study is the concurrent tracking of both protein (ELISA) and transcript (qRT‐PCR) levels across a 7‐day observation window. The two modalities showed distinct temporal patterns: qRT‐PCR demonstrated rapid early alterations and complete group separation as early as day 1, whereas ELISA protein elevations were slightly delayed but more stable. This pattern is consistent with transcription‐translation kinetics, whereby rapid microbial stimulation triggers transcription in circulating immune cells, followed by sustained synthesis and release of protein complexes. These findings suggest that transcript and protein measurements may provide complementary information on inflammatory activity and illness severity.

From a clinical biomarker perspective, traditional markers such as serum creatinine may rise only after a substantial loss of functional renal reserve, and the diagnosis of SA‐AKI is frequently delayed [3, 5]. In the present cohort, high AUC values reflect separation of groups that had already been clinically classified; therefore, they cannot establish early diagnostic performance or support claims about prediction of AKI onset [8]. Instead, the results identify the NLRP3 inflammasome axis as a biologically plausible marker of concurrent SA‐AKI and systemic disease burden [14]. Prospective inception cohorts with sampling before AKI onset are required to determine whether these markers have genuine early‐prediction value.

Several limitations should be noted. First, biomarker sampling began after sepsis and AKI classification, and the interval between sepsis onset and enrollment was not standardized, so the temporal sequence precludes conclusions about incident‐AKI prediction, early diagnosis, or causal effects. Second, this was a single‐center study with a small sample (*n* = 30) and only 13 AKI events; consequently, the observed AUC values of 1.000 are likely optimistic and may reflect complete separation. Third, the limited number of events precluded reliable multivariable adjustment, and residual confounding by baseline renal function, comorbidities, illness severity, infection source, nephrotoxic exposures, and clinical interventions [18] cannot be excluded. Fourth, the observational design cannot establish a direct causal relationship between circulating inflammasome levels and intrarenal pyroptosis. Future multicenter inception cohorts should obtain samples before AKI onset, pre‐specify clinically justified adjustment variables, use appropriately constrained or penalized models, and externally validate any candidate biomarker panel.

In conclusion, the NLRP3 inflammasome pathway is prominently activated in patients with SA‐AKI and is closely associated with the severity of both renal dysfunction and systemic illness. Circulating ASC, caspase‐1, and NLRP3 represent candidate biomarkers of concurrent SA‐AKI, with day 3 showing the strongest overall associations in this exploratory cohort. These findings support further investigation of the NLRP3 axis as a biomarker pathway and potential therapeutic target, but claims regarding early prediction or prognosis require validation in larger prospective cohorts sampled before AKI onset.

## Author Contributions

**Xiaoqian Zhang:** conceptualization, methodology, investigation, data curation, formal analysis, funding acquisition, writing – original draft, writing – review & editing. **Wang Zhang:** conceptualization, supervision, resources, writing – review & editing, funding acquisition. **Ju Guo:** methodology, investigation, resources, writing – review & editing. **Mikeriayi Aihemaiti:** investigation, data curation, formal analysis, visualization, writing – review & editing. **Yunlin Song:** investigation, data curation, writing – review & editing. All authors read and approved the final version of the manuscript.

## Ethics Statement

Exemption from full ethical review was granted by the Ethics Committee of the First Affiliated Hospital of Xinjiang Medical University; no approval or reference number was assigned (see Statement of Ethics). This study was granted an exemption from the requirement for ethics approval by the Ethics Committee of the First Affiliated Hospital of Xinjiang Medical University in accordance with local and national guidelines, because the study was a purely observational investigation involving no more than minimal risk and using only routine clinical data and residual diagnostic blood samples. In line with this exemption, no separate approval or reference number was assigned by the Committee. The study was conducted in accordance with the principles of the World Medical Association Declaration of Helsinki. Informed consent: In accordance with the exemption granted by the Ethics Committee of the First Affiliated Hospital of Xinjiang Medical University, written informed consent was not required and was not obtained; verbal informed consent was obtained from each participant or the participant's legally authorized surrogate at the time of enrollment.

## Consent

In accordance with the exemption granted by the Ethics Committee of the First Affiliated Hospital of Xinjiang Medical University, written informed consent was not required and was not obtained; verbal informed consent was obtained from each participant or the participant's legally authorized surrogate at the time of enrollment.

## Conflicts of Interest

The authors declare no conflicts of interest.

## Supporting information


**S1 Checklist:** STROBE statement checklist for cohort studies.

**S3 Table:** Complete Spearman correlation matrices (rho and two‐tailed P values) corresponding to Tables 2 and 3.

**S4 Data:** De‐identified individual‐level data underlying the findings of this study (available from the corresponding author upon reasonable request).

## Data Availability

The data that support the findings of this study are available from the corresponding author, Wang Zhang (1270903798@qq.com), upon reasonable request. Restrictions apply regarding the availability of these data, which contain identifiable clinical information collected as part of routine ICU care and were used under license for the current study; they are therefore not publicly deposited. Qualified investigators may request de‐identified datasets, together with the associated statistical code, from the corresponding author, subject to approval by the Ethics Committee of the First Affiliated Hospital of Xinjiang Medical University and execution of an appropriate data‐sharing agreement.
