# Supplementary material for: Circulating NLRP3 Inflammasome Activation Is Associated With Sepsis‐Associated Acute Kidney Injury and Disease Severity: A Prospective Pilot Study
Source: Immun Inflamm Dis. 2026 Oct 26;14(10):e70559. doi: 10.1002/iid3.70559 (PMC13710661; doi:10.1002/iid3.70559)
Supplement: Supplementary file 1 [file IID3-14-e70559-s001.docx]

STROBE Statement—checklist of items that should be included in reports of observational studies

|  | Item No. | Recommendation | Page  No. | Relevant text from manuscript |
| --- | --- | --- | --- | --- |
| **Title and abstract** | 1 | (*a*) Indicate the study’s design with a commonly used term in the title or the abstract | Page 1 | Title: Circulating NLRP3 Inflammasome Activation Is Associated with Sepsis-Associated Acute Kidney Injury and Disease Severity: A Prospective Pilot Study; Abstract Background clearly states prospective cohort design |
|  |  | (*b*) Provide in the abstract an informative and balanced summary of what was done and what was found | Page 1 | Structured abstract contains Background, Method, Results, Conclusion; describes enrollment, biomarker detection, statistical findings and study limitations |
| Introduction | | | |  |
| Background/rationale | 2 | Explain the scientific background and rationale for the investigation being reported | Pages 2–3 | SA-AKI high incidence and mortality; NLRP3 inflammasome mediates renal inflammatory injury, but human systemic expression remains unclear |
| Objectives | 3 | State specific objectives, including any prespecified hypotheses | Page 3 | Hypothesis: circulating NLRP3 inflammasome components correlate with SA-AKI presence and severity; aim to characterize longitudinal protein/mRNA expression trajectories |
| Methods | | | |  |
| Study design | 4 | Present key elements of study design early in the paper | Page 4 | Section 2.1: prospective observational pilot cohort of adult ICU sepsis patients |
| Setting | 5 | Describe the setting, locations, and relevant dates, including periods of recruitment, exposure, follow-up, and data collection | Page 4 | Single-center ICU, The First Affiliated Hospital of Xinjiang Medical University; serial blood collection on day 1, 3, 5, 7 after enrollment |
| Participants | 6 | (*a*) *Cohort study*—Give the eligibility criteria, and the sources and methods of selection of participants. Describe methods of follow-up  *Case-control study*—Give the eligibility criteria, and the sources and methods of case ascertainment and control selection. Give the rationale for the choice of cases and controls  *Cross-sectional study*—Give the eligibility criteria, and the sources and methods of selection of participants | Page 4 | Inclusion: adult sepsis patients meeting Sepsis-3; grouped by KDIGO SA-AKI diagnosis; serial blood sampling as observation follow-up |
|  |  | (*b*) *Cohort study*—For matched studies, give matching criteria and number of exposed and unexposed  *Case-control study*—For matched studies, give matching criteria and the number of controls per case | N/A | Unmatched cohort; grouping based on AKI diagnosis status, no matching performed |
| Variables | 7 | Clearly define all outcomes, exposures, predictors, potential confounders, and effect modifiers. Give diagnostic criteria, if applicable | Page 4 | Exposure: NLRP3, ASC, caspase-1, cleaved caspase-1, IL-1β, IL-18 protein/mRNA; Outcome: KDIGO-defined SA-AKI; confounders: age, BMI, APACHE II, SOFA, creatinine, eGFR |
| Data sources/ measurement | 8* | For each variable of interest, give sources of data and details of methods of assessment (measurement). Describe comparability of assessment methods if there is more than one group | Pages 4–5 | Clinical data from electronic medical records; ELISA for serum protein, qRT-PCR for mononuclear mRNA; identical testing protocols for SA-AKI and non-AKI groups |
| Bias | 9 | Describe any efforts to address potential sources of bias | Page 6 | Consecutive enrollment, unified diagnostic standards, standardized blood collection and laboratory testing to minimize measurement bias |
| Study size | 10 | Explain how the study size was arrived at | Page 4 | Single-center exploratory pilot cohort; all consecutive eligible sepsis patients enrolled, total n=30 (13 SA-AKI, 17 non-AKI); small sample limitation discussed in Discussion |

Continued on next page

| Quantitative variables | 11 | Explain how quantitative variables were handled in the analyses. If applicable, describe which groupings were chosen and why | Page 6 | Shapiro-Wilk normality test; normal data: mean±SD (t-test); skewed data: median(IQR) (Wilcoxon rank-sum); ROC optimal cutoffs calculated for exploratory discrimination only |
| --- | --- | --- | --- | --- |
| Statistical methods | 12 | (*a*) Describe all statistical methods, including those used to control for confounding | Page 6 | Chi-square/Fisher exact for categorical; t-test/Wilcoxon rank-sum for continuous; Spearman correlation; linear mixed-effects models adjusted for SOFA; exploratory ROC |
|  |  | (*b*) Describe any methods used to examine subgroups and interactions | Pages 6, 10 | Linear mixed models tested time × AKI status interaction; stratified group comparisons at each 4 sampling time points |
|  |  | (*c*) Explain how missing data were addressed | Page 6 | Participants with missing core biomarker or renal function indicators excluded from final analysis |
|  |  | (*d*) *Cohort study*—If applicable, explain how loss to follow-up was addressed  *Case-control study*—If applicable, explain how matching of cases and controls was addressed  *Cross-sectional study*—If applicable, describe analytical methods taking account of sampling strategy | N/A | All enrolled patients completed all four blood sampling time points, no loss of specimen follow-up |
|  |  | (*e*) Describe any sensitivity analyses | N/A | No formal sensitivity analyses due to limited sample size (only 13 AKI events) |
| Results | | | | |
| Participants | 13* | (a) Report numbers of individuals at each stage of study—eg numbers potentially eligible, examined for eligibility, confirmed eligible, included in the study, completing follow-up, and analysed | Page 7 | Total eligible enrolled n=30; SA-AKI n=13, non-AKI n=17; all included in final statistical analysis |
|  |  | (b) Give reasons for non-participation at each stage | N/A | No eligible patients excluded during recruitment; all consecutively admitted sepsis patients met inclusion standards |
|  |  | (c) Consider use of a flow diagram | N/A | No patient screening flowchart included; sample sizes clearly stated in text and Table 1 |
| Descriptive data | 14* | (a) Give characteristics of study participants (eg demographic, clinical, social) and information on exposures and potential confounders | Pages 7–8 | Table 1 presents age, sex, comorbidities, APACHE II, SOFA, creatinine, eGFR stratified by AKI status |
|  |  | (b) Indicate number of participants with missing data for each variable of interest | Page 7 | Minimal missing laboratory values; all incomplete cases removed prior to statistical testing |
|  |  | (c) *Cohort study*—Summarise follow-up time (eg, average and total amount) | Page 4 | Uniform 7-day observation window with serial sampling on day 1, 3, 5, 7 for all subjects |
| Outcome data | 15* | *Cohort study*—Report numbers of outcome events or summary measures over time | Pages 7–11 | 13 SA-AKI events total; longitudinal biomarker differences across four time points shown in Figure 1, Figure 2, Table 8 |
|  |  | *Case-control study—*Report numbers in each exposure category, or summary measures of exposure | N/A | Not applicable — this is a cohort study (case-control section). |
|  |  | *Cross-sectional study—*Report numbers of outcome events or summary measures | N/A | Not applicable — this is a cohort study (cross-sectional section). |
| Main results | 16 | (*a*) Give unadjusted estimates and, if applicable, confounder-adjusted estimates and their precision (eg, 95% confidence interval). Make clear which confounders were adjusted for and why they were included | Pages 9–10, Tables 4–7 | Mixed models adjusted for SOFA score; ROC curves report AUC and 95% CI; only univariate comparisons due to small sample |
|  |  | (*b*) Report category boundaries when continuous variables were categorized | Page 6, Tables 6–7 | Optimal biomarker cutoffs generated via Youden index for exploratory ROC discriminations |
|  |  | (*c*) If relevant, consider translating estimates of relative risk into absolute risk for a meaningful time period | N/A | N/A — no risk-prediction models were constructed; the study reports only exploratory bivariate associations and ROC-based discrimination of concurrent AKI status. |

Continued on next page

| Other analyses | 17 | Report other analyses done—eg analyses of subgroups and interactions, and sensitivity analyses | Pages 6, 11–12, 14 | Linear mixed-effects models tested the time × AKI status interaction (Section 3.4; Tables 4 and 5); exploratory ROC analyses (Section 3.5; Tables 6 and 7); group comparisons at each sampling time point (Section 3.6; Table 8). No formal sensitivity analyses due to the small number of AKI events (n = 13). |
| --- | --- | --- | --- | --- |
| Discussion | | | | |
| Key results | 18 | Summarise key results with reference to study objectives | pp. 15–16 (Section 4) | Circulating NLRP3 inflammasome components (protein and mRNA) were significantly elevated in SA-AKI and correlated with renal dysfunction (creatinine, eGFR) and illness severity (SOFA, APACHE II); exploratory ROC indicated strong discrimination of concurrent AKI status for ASC, caspase-1, and NLRP3 transcripts, with the strongest overall performance on day 3. |
| Limitations | 19 | Discuss limitations of the study, taking into account sources of potential bias or imprecision. Discuss both direction and magnitude of any potential bias | pp. 16–17 | Post-classification sampling precludes conclusions about incident-AKI prediction or early diagnosis; single-center design with a small sample (n = 30; 13 AKI events), with AUC values of 1.000 likely optimistic; limited events precluded multivariable adjustment; observational design cannot establish causality. |
| Interpretation | 20 | Give a cautious overall interpretation of results considering objectives, limitations, multiplicity of analyses, results from similar studies, and other relevant evidence | p. 17 (Conclusion) | Results identify the NLRP3 inflammasome axis as a candidate biomarker of concurrent SA-AKI; findings are hypothesis-generating and require validation in larger prospective cohorts sampled before AKI onset. |
| Generalisability | 21 | Discuss the generalisability (external validity) of the study results | pp. 16–17 | Single-center ICU cohort limits external validity; results require validation in larger, multicenter prospective cohorts. |
| Other information | |  | | |
| Funding | 22 | Give the source of funding and the role of the funders for the present study and, if applicable, for the original study on which the present article is based | p. 17 (Funding Sources) | Supported by the Youth Scientific Research Launch Special Fund of the First Affiliated Hospital of Xinjiang Medical University (project number 2024YFY-QKQN-78); the funder had no role in study design, data collection and analysis, decision to publish, or manuscript preparation. |

*Give information separately for cases and controls in case-control studies and, if applicable, for exposed and unexposed groups in cohort and cross-sectional studies.

**Note:** An Explanation and Elaboration article discusses each checklist item and gives methodological background and published examples of transparent reporting. The STROBE checklist is best used in conjunction with this article (freely available on the Web sites of PLoS Medicine at http://www.plosmedicine.org/, Annals of Internal Medicine at http://www.annals.org/, and Epidemiology at http://www.epidem.com/). Information on the STROBE Initiative is available at www.strobe-statement.org.
